# Determination of Chemical Compounds and Investigation of Biological Properties of *Matricaria chamomilla* Essential Oils, Honey, and Their Mixture

**DOI:** 10.3390/molecules27185850

**Published:** 2022-09-09

**Authors:** Ahmed Qasem, Hamza Assaggaf, Domenico Montesano, Zineb Khalil, Samiah Hamad Al-Mijalli, Aicha EL Baaboua, Nasreddine El Omari, Naoual El Menyiy, Saad Bakrim, Ryan A. Sheikh, Mohammed Merae Alshahrani, Ahmed Abdullah Al Awadh, Gokhan Zengin, Abdelhakim Bouyahya, Hanae Naceiri Mrabti

**Affiliations:** 1 Laboratory Medicine Department, Faculty of Applied Medical Sciences, Umm Al-Qura University, Makkah 21955, Saudi Arabia; 2 Department of Pharmacy, University of Naples Federico II, Via D. Montesano 49, 80131 Naples, Italy; 3 Laboratory of Medicinal Chemistry, Drug Sciences Research Center, Faculty of Medicine and Pharmacy, Mohammed V University in Rabat, Rabat BP 6203, Morocco; 4 Department of Biology, College of Sciences, Princess Nourah Bint Abdulrahman University, P.O. Box 84428, Riyadh 11671, Saudi Arabia; 5 Biology and Health Laboratory, Department of Biology, Faculty of Science, Abdelmalek Essaadi University, Tetouan 93000, Morocco; 6 Laboratory of Histology, Embryology and Cytogenetic, Faculty of Medicine and Pharmacy, Mohammed V University in Rabat, Taounate 34025, Morocco; 7 Laboratory of Pharmacology, National Agency of Medicinal and Aromatic Plants, Taounate 34025, Morocco; 8 Geo-Bio-Environment Engineering and Innovation Laboratory, Molecular Engineering, Biotechnologies and Innovation Team, Polydisciplinary Faculty of Taroudant, Ibn Zohr University, Agadir 80000, Morocco; 9 Biochemistry Department, Faculty of Science, King Abdulaziz University, Jeddah 21589, Saudi Arabia; 10 Department of Clinical Laboratory Sciences, Faculty of Applied Medical Sciences, Najran University, Najran 61441, Saudi Arabia; 11 Department of Biology, Science Faculty, Selcuk University, Konya 42250, Turkey; 12 Laboratory of Human Pathologies Biology, Department of Biology, Faculty of Sciences, Mohammed V University in Rabat, Rabat BP 6203, Morocco; 13 Laboratory of Pharmacology and Toxicology, Bio Pharmaceutical and Toxicological Analysis Research Team, Faculty of Medicine and Pharmacy, University Mohammed V in Rabat, Rabat BP 6203, Morocco

**Keywords:** *Matricaria chamomilla* L., essential oils, antimicrobial activity, antidiabetic activity, anti-inflammatory effect, antioxidant effect

## Abstract

This exploratory investigation aimed to determine the chemical composition and evaluate some biological properties, such as antioxidant, anti-inflammatory, antidiabetic, and antimicrobial activities, of *Matricaria chamomilla* L. essential oils (EOs). EOs of *M. chamomilla* were obtained by hydrodistillation and phytochemical screening was performed by gas chromatography–mass spectrophotometry (GC-MS). The antimicrobial activities were tested against different pathogenic strains of microorganisms by using disc diffusion assay, the minimum inhibitory concentration (MIC), and minimum bactericidal concentration (MBC) methods. The antidiabetic activity was performed in vitro using the enzyme inhibition test. The antioxidant activity of EOs was tested using the free radical scavenging ability (DPPH method), ferrous ion chelating (FIC) ability, and *β*-carotene bleaching assay. The anti-inflammatory effects were tested in vivo using the carrageenan-induced paw edema method and in vitro using the inhibition of the lipoxygenase test. The analysis of the phytochemical composition by GC-MS revealed that camphor (16.42%) was the major compound of EOs, followed by 3-carene (9.95%), *β*-myrcene (8.01%), and chamazulene (6.54%). MCEO, honey, and their mixture exhibited antioxidant activity against the DPPH assay (IC_50_ ranging from 533.89 ± 15.05 µg/mL to 1945.38 ± 12.71 µg/mL). The mixture exhibited the best radical scavenging activity, with an IC_50_ of 533.89 ± 15.05 µg/mL. As antidiabetic effect, EO presented the best values against α-glucosidase (265.57 ± 0.03 μg/mL) and α-amylase (121.44 ± 0.05 μg/mL). The EOs and honey mixture at a dose of 100 mg/kg exhibited a high anti-inflammatory effect, with 63.75% edema inhibition after 3 h. The impact of EOs on the studied species showed an excellent antimicrobial (*Staphylococcus aureus* ATCC 29213 (22.97 ± 0.16 mm)), antifungal (*Aspergillus niger* (18.13 ± 0.18 mm)) and anti-yeast (*Candida albicans* (21.07 ± 0.24 mm) effect against all the tested strains. The results obtained indicate that the EOs of *M. chamomilla* could be a potential drug target against diabetes, inflammation and microbial infections; however, further investigations to assess their bioactive molecules individually and in combination are greatly required.

## 1. Introduction

The therapeutic benefits of plants have been proven for centuries. Many disease states have been successfully remedied using plant-derived medicines. These remedies are employed in the form of concoctions or concentrated extracts of plants, without separating the active components. Nevertheless, there are many health problems worldwide with disorders such as diabetes, hypertension, chronic inflammation, microbial infections, degenerative diseases, and cancer for which contemporary medicine is still trying to find appropriate treatments [1]. Indeed, the use of traditional medicine has been eclipsed by modern medicine as a way to cure and manage human illnesses [2]. However, in recent decades, the use of herbal medicines in promoting health and treating conditions has increased in several countries, including developing societies [3]. Natural products can be considered as sources of therapeutic ingredients for discovering innovative drugs [4,5,6]. They could, therefore, constitute a tool for identifying molecules with several pharmacological and biological characteristics, which will be available and effective for the management of different human and animal pathologies [7,8,9,10]. Regardless, the problem of drug discovery is multifaceted and needs the assessment of multiple endpoints of natural and synthetic agents, such as pharmacokinetics, safety, and efficacy, when screening drug compounds [1,11].

Currently, it is well known that natural molecules, in particular those derived from medicinal plants, have already demonstrated interesting outcomes, due to their antimicrobial actions against different pathogenic strains, their anti-diabetic effects in vitro and *in vivo*, and their anti-inflammatory properties through different molecular and cellular mechanisms [12,13,14,15].

*Matricaria chamomilla* L. (chamomile) is a popular herbal medicine from the Asteraceae family that is widely distributed throughout the world [16]. The plant can be found in North Africa, North and South America, Australia, New Zealand, and Asia. It is also cultivated in France, Germany, Brazil, Yugoslavia, Hungary, and Russia [16]. It is nowadays a very appreciated and highly used medicinal plant in popular and traditional medicine for the treatment of a certain number of pathologies, especially diabetes and chronic inflammation, but also can be used for treating colic, flatulence, hysteria and intermittent fever [17].

*M. chamomilla* contains a broad spectrum of secondary metabolites and active compound classes. Flavonoids, sesquiterpenes, polyacetylenes, and coumarins are identified as the major components of chamomile. Among the bioactive phenolic compounds present in chamomile extracts, we found chlorogenic acid and caffeic acid, luteolin and luteolin-7-O-glucoside, quercetin and rutin, apigenin and apigenin-7-O-glucoside, and naringenin [16]. As chamomile is an excellent natural source of medicinal products, this study was designed to highlight the phytochemical composition and valorize the EOs (terpene compounds) of this plant against different biological systems, namely antioxidant, antidiabetic, anti-inflammatory, and antimicrobial effects.

## 2. Materials and Methods

### 2.1. Essential Oil Origin

The leaves of *M. chamomilla* were collected from the Taza region (north-west of Morocco) in April 2021. The plant material was identified and authenticated (voucher specimen: RAB 104230) from the Department of Botany and Plant Ecology of the Scientific Institute of Rabat, University of Mohammed V Rabat, Morocco. After collection, the leaves were then dried and *M. chamomilla* essential oil (MCEO) was extracted by hydrodistillation.

### 2.2. Honey Samples

Samples of honey were collected from natural hives in the Taza region (north-west of Morocco) during the month of April. Indeed, during this period, the hives become rich with honey. The samples were kept at room temperature and the different analyses were performed within a time period that did not exceed three months from the date of collection.

### 2.3. Physicochemical Properties of M. chamomilla Honey

Physicochemical properties of *M. chamomilla* honey have been estimated by many methods. All tests were performed as described by Mekkaoui et al. [18].

### 2.4. Chemical Analysis of Volatile Compounds

The chemical components of MCEO were determined using GC/MS analysis, as described previously [18]. Indeed, a Hewlett–Packard (HP6890) GC instrument coupled with an HP5973 MS and equipped with a 5% phenylmethyl silicone HP-5MS capillary column (30 m × 0.25 mm × film thickness 0.25 μm) was used in the GC analysis. The used column temperature increased from 50 °C for 5 min to 200 °C, with a 4 °C/min rate. Helium with a 1.5 mL/min flow rate and split mode (flow: 112 mL/min, ratio: 1/74.7) was the carrier gas used. The hold time was 48 min, while the temperature of the injector and detector was 250 °C.

The machine was led by a computer system type “HP ChemStation”, which managed the functioning of the machine and allowed us to follow the evolution of the chromatographic analyses. Diluted samples (1/20 in methanol) of 1 μL were injected manually. In addition, 70 eV ionization voltage, 230 °C ion source temperature, and 35–450 (*m*/*z*) scanning range were the MS operating conditions. Finally, the qualitative quantification of the different compounds was based on the percent area of each peak of the sample compounds and confirmed by reference to their MS identities (Library of NIST/EPA/NIH MASS SPECTRAL LIBRARY version 2.0, built 1 July 2002).

### 2.5. Antioxidant Activity

The estimation of the antioxidant activity was carried out using three complementary methods.

#### 2.5.1. Free Radical Scavenging Ability (DPPH Method)

The ability of EOs to scavenge the DPPH radical was measured using the method of Brand-Williams [19]. Aliquots (40 µL) of samples were added to 3 mL of DPPH solution (6 × 10^−5^ mol/L) and the absorbance was determined at 515 nm after 60 min.

#### 2.5.2. Determination of Ferrous Ion Chelating Ability (FIC Assay)

Firstly, solutions of MCEO at different concentrations were mixed with the FeSO_4_·7H_2_O solution (2 mM), and then ferrozine solution (5 mM) was added to the vial to initiate the reaction. After 10 min, the optical density was read at 562 nm. The FIC was calculated according to the following equation:

FIC% = [(Ac − As)/Ac] × 100

Ac is the absorbance of the reference (contains ferrous sulfate and ferrozine) and As is the absorbance of the EO (ferrous sulfate and ferrozine with EOs of *M. chamomilla* solution).

#### 2.5.3. β-. Carotene Bleaching Assay (BCB)

The BCB assay was carried out as previously reported by Belmehdi et al. [20]. An emulsion of β-carotene, linoleic acid, and tween 40 was prepared. A specific volume of the emulsion was added to EOs of *M. chamomilla* solution and the absorbance was immediately read at 470 nm, with intervals up to 180 min. Using the following formula, the antioxidant activity (AA) was evaluated in terms of the bleaching of β-carotene percentage:

AA% = [1 − (As 0 − As t)/(Ac 0 − Ac t)] × 100

where As 0 and Ac 0 are the respective absorbances of MCCO and control at zero time, As t and Ac t are the respective absorbances of EOs of *M. chamomilla* and control after 180 min.

#### 2.5.4. 5-Lipoxygenase (5-LOX) Inhibition Assay

The lipoxygenase inhibitory activity of *M. chamomilla* was evaluated by following the linoleic acid oxidation at 234 nm, according to a previous published method [21]. Briefly, 20 µL of oil and 20 µL of 5-LOX from Glycine max (100 U/mL) were pre-incubated with 200 µL of phosphate buffer (0.1 M, pH 9), at room temperature for 5 min. The reaction was started by the addition of 20 µL of linolenic acid (4.18 mM in ethanol) and followed for 3 min at 234 nm. The results correspond to the mean ± SD of three independent assays, each performed in triplicate. Quercetin was used as a positive control.

### 2.6. In Vivo Anti-Immflamatory

The anti-inflammatory effects were studied using the carrageenan-induced paw edema method previously described by Musa G.Rege et al. [22]. Briefly, Wistar rats (150 to 180 g) were randomly divided into eight groups (*n* = 6). The animals fasted for 18 h prior to testing. The groups of rats were orally administered with different concentrations of the studied drugs (MCEO, *M. chamomilla* honey, and a mixture of the EO and honey (1:1) (50 and 100 mg/kg). The control group receives distilled water, while the last group received indomethacin (10 mg/kg) as the reference drug. After 60 min, all rats were injected subcutaneously with carrageenan solution (0.05 mL of 1% carrageenan suspended in 0.9% NaCl) into the subplantar region of the left hind paw. The paw volumes of the rats were recorded using a LE 7500 digital plethysmometer, controlled by SeDaCOM software, just before the carrageenan injection *(V_0_*), then at 1 h, 3 h, and 6 h after the carrageenan injection (*V_t_*). The anti-inflammatory effect is calculated using the following equation:

$$\%\;inhibition=\frac{\left(V_{t}-V_{0}\right)control-\left(V_{t}-V_{0}\right)treated\;group}{\left(V_{t}-V_{0}\right)\;control}\times 100$$

### 2.7. Antimicrobial Activity

#### 2.7.1. Tested Micro-Organisms

The antibacterial activity was evaluated against the following six bacterial strains, representing Gram-positive and Gram-negative bacteria: Escherchia coli (*E. coli*) ATCC 25922, *Proteus mirabilis* (*P. mirabilis*) ATCC 25933, *Salmonella* Typhimurium (*S. Typhimurium*) ATCC 700408, (*B. subtilis*) ATCC 6633, *Staphylococcus aureus* (*S. aureus*) ATCC 29213, and *Listeria monocytogenes* (*L. monocytogenes*) ATCC 13932.

#### 2.7.2. Disc Diffusion Assay

The antimicrobial activity of MCEO, honey and their mixture against the tested microorganisms was investigated using the disc diffusion method, according to the previously published protocols [23,24]. Briefly, the culture suspension was inoculated by swabbing on optimal culture media (Mueller–Hinton agar for bacteria and Sabouraud agar for yeast and fungi) and then the MCEO oil (mixed with 5% of DMSO), honey, or the mixture of MCEO and honey were deposited on each plate. Chloramphenicol (30 µg) was used as a positive control for bacteria and nystatin (100 I.U.) was used as a positive control for yeast and fungi, while DMSO (10 µL; 5%) was used as a negative control. The bacterial plates were incubated at 37 °C for 24 h, the yeast plates were incubated at 25 °C for 48 h, and the fungi plates were incubated at 25 °C for 72 h. After incubation, the inhibitory diameters were measured in millimeters and the results were expressed as means ± standard deviation of the three replicates.

#### 2.7.3. Determination of MIC

The MIC corresponds to the minimum concentration of samples that can inhibit the growth of microorganisms. In fact, the determination of MIC against bacteria and yeast was performed according to the protocol described previously [25], with some modifications, in which Mueller–Hinton broth (Biokar, Beauvais, France) was used for bacteria and Sabouraud broth (Biokar, Beauvais, France) was used for yeast. The incubation was conducted at 37 °C for 24 h for bacteria and 25 °C for 48 h for yeast. However, the determination of MIC against the fungi was conducted by the gradient plate method according to the protocol described previously [26]. The chloramphenicol was used as a positive control for bacteria, while nystatin was used for yeast and fungi.

#### 2.7.4. Determination of MBC

Minimum bactericidal concentration (MBC) corresponds to the minimum concentration of samples that can kill the bacteria. The same microdilution experiment derived from the determination of MIC was used. After the incubation, 5 μL of each tube that did not present visible growth was subcultured on tryptone soy agar (Biokar, Beauvais, France) and incubated at 37 °C for 24 h, and the lowest concentration that did not present any growth on media was considered as the MBC [27].

## 3. Results and Discussion

### 3.1. Physicochemical Properties of M. chamomilla Honey Sample

The physicochemical properties of the studied honey sample, including color, moisture, pH, free acidity, hydroxymethylfurfural (HMF), lactone acidity, electrical conductivity, density and ash content, are shown in Table 1. The moisture content is an important parameter to determine the quality of honey, as it is closely linked to climatic conditions, harvest seasons, honey storage, and nectar sources used by bees. Since the moisture content in *M. chamomilla* honey was 11.04 ± 0.02%, which was within the internationally recommended parameters (≤20%) for commercial honey quality [28,29]. These results suggest the appropriate handling and storage of the honey. The analyzed honey sample showed acidic pH values (4.02 ± 0.01). Similar values were reported for other Moroccan honey samples [30,31,32,33,34]. This acidity is due to the presence of many organic acids, such as gluconic acid and their lactones and esters. This parameter has an important effect on conservation, due to its capacity to limit and inhibit the growth and proliferation of several microorganisms, making it an important factor for honey texture and stability [35]. Free acidity, established by the Codex Alimentarius and European Community regulations, requires, in general, no more than 50 milliequiv acid/kg [28,29]. The free acidity of the honey sample in this study was 22.78 ± 0.01 meq/kg, while the lactone acidity value (considered as the acidity reserve when honey becomes alkaline) was 3.01 ± 0.05 meq/kg. This acidity is similar to other kinds of honey from other botanical and geographical origins and is due to the presence of organic acids and inorganic ions, such as phosphate [36,37,38].

The analyzed *Chamomilla* honey showed an electrical conductivity value greater than 0.48 ± 0.01 mS/cm, suggesting that the honey sample is from nectar honey, which is supported by the total ash content of less than 0.6%. The color of honey is related to the phenol and flavonoid content, mineral and pollen content and may be influenced by the storage and processing conditions. In this study, the honey sample presented an extra light amber color, which confirmed that *Chamomilla* honey has a higher content of these compounds.

The HMF content, an indicator of honey freshness that increases during processing and/or aging, is influenced by several factors, such as temperature, pH, storage conditions, and the concentrations of metallic ions, such as manganese, zinc, magnesium, and iron (II)-presenting honey. The analyzed sample has a level lower than the maximum established by international standards (<40 mg/kg) [29].

### 3.2. Chemical Composition MCEO

Regarding the analysis of the chemical composition of MCEO, it was carried out by the GC-MS method (Table 2). In total, 49 molecules were identified in our study, with percentages ranging from 0.11 to 16.42%. Indeed, camphor (16.42%) was the major compound of our EO, followed by 3-carene (9.95%), *β*-myrcene (8.01%), and chamazulene (6.54%). Similarly, other main molecules were determined with percentages of 5.93, 5.34, 4.23% and 3.04% for 2,4,5-tetramethyl-1,3-cyclopentadiene, 3,6-dihydrochamazulene, *α*-phellandrene, and *β*-sesquiphellandrene, respectively. On the other hand, many research works have investigated the chemical composition of this plant, as well as that of another species of the same family called *Matricaria recutita*. Indeed, concerning chamazulene, our results perfectly corroborate those obtained from *M. chamomilla* var. *Chamomilla* flower EO, in a study performed by Mahdavi et al. (2020) with a percentage of 6.46% [39]. Previous studies have revealed the great diversity in the chemical composition of the different extracts and EOs of this species [40,41,42,43].

This diversity has been associated with several factors, in particular the harvest region. In 2002, two Iranian researchers evaluated the phytochemical profile of this plant collected from three different Iranian cities (Tehran, Kazeroon, and Hammadan) during different periods [44]. Therefore, they detected several phytochemicals, including chamazulene with a concentration of 2.6%. Additionally, Ashnagar and collaborators separated and identified three main molecules, namely chamazulene (detected in our study), bisabololoxid A, and bisabolonoxid from *M. chamomilla* harvested in the northwest of Khuzestan, Iran [45]. This province, along with others in the same country, was the subject of a more recent study to compare the volatile compounds of twelve different oil samples from *M. chamomilla* growing in Iran [46]. The authors found a striking correlation between MCEO phytochemical profiles and geographic factors.

This correlation has already been recorded by Satyal and colleagues, who observed a strong diversity in the chemical composition of Nepalese chamomile EO [47]. It was discovered that in addition to these geographical factors, others are also involved in the variation in MCEO, such as the differences in irrigation regimes [48], the application of certain trace elements [49], the choice of extraction temperature [50] and method [51], the drying conditions [52], and agronomic interventions [53].

### 3.3. Antioxidant Activity

The antioxidant activity of MCEO, honey, and their mixture was analyzed using three different methods (DPPH, FIC ability, and BCB assay). The results are shown in Table 3 and the IC_50_ values were calculated to compare these results to those of BHT, which was used as the reference standard. All samples had the capacity to reduce the stable violet DPPH radical to yellow DPPH-H, with 50% of the reduction values (IC_50_) ranging from 533.89 ± 15.05 µg/mL to 1945.38 ± 12.71 µg/mL. The mixture exhibited the best radical scavenging activity, with an IC_50_ of 533.89 ± 15.05 µg/mL and the best ferrous ion chelating ability, with an IC_50_ value of 713.69 ± 03.02 µg/mL. The IC_50_ value of synthetic antioxidant BHT was 14.24 ± 1.32 µg/mL in DPPH and 42.12 ± 0.07 µg/mL in FIC ability, indicating better antioxidant activity of BHT compared to the tested samples. The BCB assay honey sample presented the best activity with a percentage of 745.54 ± 8.03%, compared with the other samples and the BHT used as a reference standard. Several studies on honey indicate that the antioxidant activity of honey is due to the presence of the mentioned bioactive compounds, such as polyphenols and flavonoids and varies widely, depending on floral source and geographical origin [31,54]. Furthermore, no scientific exploration has measured the antioxidant ability of *M. chamomilla* honey but the previous antioxidant activity of MCEO showed the best antioxidant properties, with an IC_50_ value of 2.20 mg/mL, by the DPPH method [55]. In another work, the antioxidant activity of MCEO was studied by DPPH and β-carotene/linoleic acid methods, and the IC_50_ value was determined as 5.63 ± 0.20 mg/mL [56]. In the BCB assay, MCEO gave the best inhibition result, with an inhibition rate of 82.5%, supporting the antioxidant activity of MCEO [57]. The antioxidant activity of MCEO was attributed also to their terpenes, such as *α*-pinene, *β*-pinene, *β*-myrcene and *γ*-terpinene, which are known to have good antioxidant properties [43,58,59]. According to these results, the combination between *M. chamomilla* honey and MCEO presented the best antioxidant capacity and also had the capacity to inhibit lipid peroxidation, which may be due to the synergistic effects of their compounds. These results give this mixture an important potential in combating oxidant damage and can be potentially used as a safer alternative to synthetic antioxidants in the pharmaceutical and food industries.

### 3.4. Anti-Diabetic Activity

α-Glucosidase and α-amylase are key digestive enzymes that are implicated in the intestinal metabolism of carbohydrates and lipids [60]. Therefore, inhibition and suppression of these enzymes is a promising therapeutic strategy in the management of type 2 diabetes mellitus (T2DM). In this regard, the inhibitory capacity of α-glucosidase and α-amylase of MCEO, *M. chamomilla* honey and their mixture was evaluated (Table 4). The results showed that the EO presented the best values against α-glucosidase (265.57 ± 0.03 μg/mL) and α-amylase (121.44 ± 0.05 μg/mL), compared to the honey sample (1351.02 ± 0.01 μg/mL and 845.31 ± 0.02 μg/mL, respectively) and their mixture (981.44 ± 0.05 μg/mL and 757.23 ± 0.02 μg/mL, respectively). However, its activity remained lower than that of acarbose as a standard reference for α-glucosidase (199.53 ±1.12 μg/mL) and more important than that of acarbose for α-amylase (396.42 ± 5.16 μg/mL). Moreover, our results are in agreement with those obtained in several works (in vivo and in vitro) that evaluated the antidiabetic effect of *chamomilla* plant extracts. Indeed, *M. chamomilla* ethanolic extract demonstrated anti-glycation properties with an IC_50_ of 264.2 µg/mL for lipase inhibition activity [61]. Moreover, Villa-Rodriguez et al. [62] showed that the *M. chamomilla* extract and isolated apigenin, apigenin-7-O-glucoside, cis and trans-2-hydroxy-4-methoxy cinnamic acid glucosides exhibited concentration-dependent inhibition of α-amylase and maltase [62]. On the other hand, Najla and collaborators revealed that oral administration of *M. chamomilla* leaves’ water extract at a dose of 200 mg/kg/day significantly reduced blood glucose levels and increased plasma insulin in streptozotocin-diabetic rats [63]. Using the same animal model, the same results were recorded by Ramadan et al., [64] which have demonstrated that the administration of the water extract of *M. chamomilla* leaves at a concentration of 100 mg/kg body weight significantly reduced blood glucose levels in diabetic animals and elevated the levels of serum insulin and C-peptide. The antidiabetic activity of EOs was certainly due to their terpenes, such as α-pinene, *β*-myrcene, *α*-thujone and *γ*-terpinene [43,59] that are known to have good antidiabetic activity. Indeed, α-pinene was found to be able to reduce fasting blood glucose levels in alloxan-induced diabetic mice after 2 and 24 h of treatment [65]. Moreover, oral administration of *α*-thujone (60 mg/kg/day) has been shown to be able to decrease plasma glucose levels in STZ-induced diabetic rats [66]. From this, and the antioxidant properties already confirmed in our work, it can be deduced that *M. chamomilla* can be used for controlling blood glucose levels and the oxidative stress that accompanies diabetes.

### 3.5. In Vivo Anti-Inflammatory Activity

Immunomodulators are natural or synthetic substances that regulate the type, duration, and intensity of innate or adaptive immune responses. Because of excessive side effects of anti-inflammatory synthetic drugs on human health, high cost, and drug resistance of chemical immunomodulators, researchers have now focused on naturally originated agents as a specific, safe, and inexpensive treatment [67]. Indeed, in this work, we evaluated the anti-inflammatory activity of MCEO, *M. chamomilla* honey and their mixture on carrageenan-induced acute inflammation by measuring the increased paw volume of the rats at different time periods (1, 3 and 6 h) (Table 5). Our results showed that the *MCEO* and honey mixture at a dose of 100 mg/kg exhibited maximum anti-inflammatory activity, with 63.75% and 77% edema inhibition after 3 h and 6 h, respectively. The results were comparable with the reduction produced by 10 mg/kg of indomethacin, a standard drug, at 3 h and 6 h, which presented inflammation inhibition values of 56.25% and 69%, respectively. Our data are similar to previous studies that demonstrate the anti-inflammatory activity of the extracts and MCEO. For instance, Wu et al. [68] showed that MCEO and aqueous extract could significantly inhibit pedal swelling induced by carrageenan in rats by decreasing the concentration of PGE2 and NO. Elmallh et al. [69] revealed that the aqueous chamomile extracts significantly decreased the paw thickness of rats after induction of pedal inflammation, as compared to the positive group. Ortiz et al. [70] demonstrated also that *M. chamomilla* ethanolic extract (MCE) was able to inhibit the cyclooxygenase (COX) enzyme *in silico.* This activity could be attributed to the presence of chemical constituents, such as flavonoids in honey, which have been widely reported to inhibit the COX and lipooxygenase pathways of arachidonate metabolism [71]. They have been shown also to inhibit the release of proinflammatory cytokines TNF-α and IL-1β, as well as down-regulate the expressions of inducible nitric oxide synthase (iNOS) and the production of reactive oxygen species (ROS). Moreover, several other mechanisms are involved, including the activation of the nuclear factor erythroid 2-related factor 2/antioxidant response element pathway and down-regulation of the nuclear factor kappa B pathway (NF-kB). On the other hand, flavonoids can also modulate gene expression of numerous inflammatory factors via down-regulation of epigenetic transcriptional control of these genes [72,73]. It is also due to MCEO’s constituents, such as α-pinene, β-pinene, β-myrcene, and *γ*-terpinene [43,58,59] that have been widely investigated for their anti-inflammatory effects [74,75] [76].

### 3.6. In Vitro Anti-Inflammatory and Dermatoprotective Activity

The inhibition of tyrosinase activity is an important strategy for skin protection. Indeed, the tyrosinase inhibitory method is the most used in vitro method today for revealing the dermatoprotective activity of medicinal plant products. On the other hand, the inhibition of lipoxygenase is the most used test to evaluate the anti-inflammatory activity in vitro. In this work, the ability of MCEO, honey and their mixture to protect the skin was assessed by its tyrosinase inhibitory property and anti-inflammatory effect by the 5-LOX inhibition assay. The results of tyrosinase and 5-LOX inhibition by all samples are expressed as IC_50_ (Table 6). As listed, MCEO presented the best activity, with an IC_50_ value of 41.11 ± 0.03 µg/mL compared to honey (81.53 ± 0.01 µg/mL) and their mixture (57.32 ± 0.03 µg/mL) but was lower than that of the standard, quercetin (IC_50_ = 39.28 ± 0.02 µg/mL). Moreover, the anti-inflammatory effect of EO (IC_50_ = 1.58 ± 0.02 µg/mL) was also higher than that of the other samples and lower compared with quercetin (IC_50_ = 1.02 ± 0.01 µg/mL). Our results are in agreement with those obtained by Jo and colleagues [77], who examined, in vitro, the whitening effect of *M. chamomilla L*. extract using tyrosinase inhibitory assay. The authors recorded important anti-tyrosinase activity in a concentration-dependent manner of the ethanol extract. Furthermore, Danciu et al. [78] showed that methanolic extract of *M. chamomilla* showed good inhibition of lipoxygenase, with an EC_50_ value of 166.32 mg/mL. From this, it can be deduced that preparations based on *M. chamomilla* can be used as sunscreen formulations to protect the skin against sunburn and to slow down skin aging and inflammation diseases.

### 3.7. Antimicrobial Activity

In vitro, the Moroccan MCEO, honey and their mixture together were screened for their antimicrobial activity using disk diffusion assay against Gram-positive, Gram-negative pathogenic bacteria, yeast and fungus and the reference species and the results are presented in Table 7. The obtained inhibitory diameters, expressed in millimeters, showed a variation in results in terms of an examined sample of *M. chamomilla*. As confirmed, the EO of the study species exhibited an excellent antimicrobial, antifungal and anti-yeast effect against all the tested strains, compared to the *M. chamomilla* honey samples. The measured inhibition zones of MCEO registered very potent activity, in which *Staphylococcus aureus* ATCC 29213 (22.97 ± 0.16 mm), *Listeria monocytogenes* ATCC 13,932 (22.10 ± 0.13 mm), *Candida albicans* (21.07 ± 0.24 mm) and *Proteus mirabilis* ATCC 25933 (19.33 ± 0.16 mm) were the most sensitive strains, followed by *Aspergillus niger* (18.13 ± 0.18 mm), *Escherichia coli* ATCC 25922 (16.87 ± 0.16 mm), *Salmonella* Typhimurium ATCC 700408 (16.23 ± 0.09 mm) and *Trichophyton rubrum* (15.87 ± 0.16 mm).

Moreover, our findings indicated a mild antimicrobial activity of *M. chamomilla* honey against *L. monocytogenes*, *S. aureus*, *P. mirabilis, C. albicans* and *E. coli* corresponding to inhibition diameters ranging from 11.97 ± 0.11 mm to 13.90 ± 0.13 mm, respectively. One must note that the honey was not active on *P. aeruginosa* and *T. rubrum* and *A. niger* were represented by the smallest inhibitory zone of *M. chamomilla* honey of 8.10 ± 0.01 mm, respectively. It is worthwhile noting that the mixture exhibited an additive effect between both samples of this plant. All examined bacteria, fungus and yeast were responsive and sensitive to chloramphenicol and nystatin, respectively (Table 7).

Using broth microdilution and gradient plate methods, the measurements of MIC and MBC of MCEO and *honey* and their mixture were performed for bacterial, yeast and fungal strains. As recapitulated in Table 8, the values of inhibitory concentrations registered for MCEO showed higher MICs than those obtained in *MCEO* honey and their association in all tested microorganisms. Indeed, *S. aureus* and *L. monocytogenes* indicated the lowest MIC values of MCEO (0.25% (*v*/*v*)), followed by *E. coli*, *P. mirabilis* and *C. albicans*, with an MIC of *M. chamomilla* 0.5% (*v*/*v*). An MIC of 1% (*v*/*v*) of MCEO was observed in *S. Typhimurium* and *A. niger*, whereas 2% (*v*/*v*) was the MIC marked in *T. rubrum* inoculums in the same aforementioned sample type (Table 8). The highest MICs identified were in E. coli, *P. mirabilis, S. Typhimurium, A. niger* for an MIC of 4% (*v*/*v*), pursued by *T. rubrum* and *P. aeruginosa* for an MIC that ranged between 4 and 8% (*v*/*v*) in *M. chamomilla* honey samples. Similar to the disk diffusion test, the findings of MIC in the combination with honey and MCEO showed mutual interaction against the investigated pathogens.

The recorded MIC values indicate that a bactericidal effect of MCEO was only detected in Gram-positive bacterial suspensions of *S. aureus* and *L. monocytogenes* (MIC = MBC = 0.25% (*v*/*v*)) and *S*. *Typhimurium* (MIC = MBC = 1% (*v*/*v*)). However, a double quantity of MIC was the efficient dose to kill other bacteria, in which the MBC was equal to 1% and > 8% in *E. coli*, *P. mirabilis* and *P. aeruginosa* by MCEO, respectively. Likewise, the same doses were measured in *M. chamomilla* honey and the combination between MCEO and *M. chamomilla* honey (Table 8).

Numerous investigations on aromatic and medicinal plants agreed that EOs exhibited strong inhibitory action on pathogenic microbes [24,79,80]. Nonetheless, the antimicrobial activity findings of the present work revealed a variation in response degree according to the strains’ origin (bacteria, fungus and yeast) and type of tested specimen of *M. chamomilla* (EOs, honey and their mixture). This is the first research paper in vitro about Moroccan *M. chamomilla* honey and its association between EOs and honey of *M. chamomilla*. In reality, a very powerful antimicrobial effect from MCEOs by both disk diffusion and broth microdilution techniques were cited, where the measured diameter was between 22.97 ± 0.16 mm and 15.87 ± 0.16 mm, and MIC values ranged from 0.25% (*v*/*v*) to 2% (*v*/*v*), respectively, against the studied microorganisms, except *P. aeruginosa*. Moderate *activity* was observed in the *M. chamomilla* mixture of honey and EOs (inhibitory diameter was between 13.90 ± 0.13 mm and 8.10 ± 0.01 mm vs. MIC ranging from 1% (*v*/*v*) to >8% (*v*/*v*)). However, a weak *antimicrobial* impact was displayed in *M. chamomilla* honey (inhibitory diameter was between 13.90 ± 0.13 mm and 8.10 ± 0.01 mm vs. MIC ranging from 2% (*v*/*v*) to >8% (*v*/*v*)).

Few studies were consistent with our findings, such as the outcomes of the Brazilian team conducted by Silva et al. [81]. These researchers confirmed, in their comparative antibacterial and phytochemical analysis of crude extracts, that MCEOs showed the best antimicrobial agents, in which *S. aureus* (MIC_90%_ = 1.2 mg/mL) was significantly (*p* ≤ 0.05) more susceptible compared to *E. coli* (MIC_90%_ = 28.2 mg/mL) [81]. Das et al. [82] assessed antimicrobial activity by the formulation of a pickering nanoemulsion of MCEO and confirmed the great antibacterial action on *P. aeruginosa* PMC 103, S. aureus ATCC 29,213 and E. coli PMC 201 for an MIC_90_ equal to 1.02 µg/mL, 1.06 µg/mL and 2.19 µg/mL, respectively. Moreover, the extraction and valorization of the antibacterial activity of wild chamomile from the Taounate province, Morocco, stated comparable relevant inhibitory diameters and MICs [83].

Contrary to the impressive antimicrobial action exposed in this investigation, most published data have reported the powerless antimicrobial activity of MCEOs across the entire world. The disc-diffusion and broth microdilution tests carried out by Soković et al. [84] demonstrated that MCEOs extracted from the aerial plant in Serbia were ineffective against the human strain of *P. mirabilis* and reference strains of *P. aeruginosa* ATCC 27,853 (0 mm vs. *MIC = MBC =* 10.0 and 15.0 μg/mL, respectively). In the same way, no effects were recorded in *S. aureus* ATCC 25923, *E. coli* ATCC 0157:H7, *S. typhimurium* ATCC 13,311 or *L*. *monocytogenes* inoculums, where the inhibitory diameter, MIC and bactericidal doses ranged from 8.0 mm to 10.0 mm, and from 5.0 μg/mL to 10.0 μg/mL [84]. As proven by Herman et al., [85] and Mekonnen et al. [86], and Niknam et al. [87] through the use of the agar diffusion method, the methanol fraction of M. chamomilla flower extract (50 mg/mL) showed weak antibacterial effect against S. aureus ATCC 6538p (1.3  ±  0.3 mm) and *P. aeruginosa* ATCC 9027 (0.3  ±  0.3 mm). A higher MIC was noticed toward the methanol fraction of flower M. chamomilla extract on S. aureus ATCC 6538p (62.5 μg/mL) and (500 μg/mL) *P. aeruginosa* ATCC 9027.

Based on the evaluated antifungal activity from the M. chamomilla flower EOs on A. niger, Tolouee et al. [88] marked a dose-dependent effect with maximum growth inhibition of approximately 92.50% of A. niger. In addition to this, the authors described other morphological changes, such as retardation in conidial production of the fungus species, observed by transmission electron microscopy due to direct penetration of MCEOs through the fungal plasma membrane [88]. However, Mekonnen et al. [86] have proven that *Trichophyton* spp., and *Aspergillus* spp., donated by the Ethiopian Public Health Institute, were resistant to EOs extracted from matured flower heads of *M. chamomilla*. In addition, several common molds found in cakes and bakery products, including *A. niger,* were significantly killed after treating these with chamomile EO at 0.15% [89]. Their findings allowed them to increase the shelf-life of food products without the use of synthetic agents [89]. Das et al. [82] concluded that the nanoemulsion of chamomile EOs demonstrated relevant antifungal activity, especially on C. albicans ATCC 1001 for an MIC_90_ equal to 2.65 µg/mL. Concerning the data about the antimicrobial effect of M. chamomilla honey or their association with EOs, no published paper was found while reporting this work. Instead, the additive effect shown in the combination of MCEO and honey confirmed the promising antimicrobial activity of MCEO against the most abundant pathogens assayed in this report.

The comparison of literature data about the antimicrobial effect of MCEOs have illustrated considerable levels of divergence [83,86,89,90]. In reality, numerous molecules were described as major phytochemicals of MCEO, in particular, E-β-farnesene (34.61 ± 3.79%) in the United Kingdom [91], α-bisabolol oxide B (51.428%), chamazulene/azulene (17.688%), trans-β-farnesene (6.953%) in Ethiopia (Mekonnen et al. [86], α-bisabolol (56.86%), trans-trans-farnesol (15.64%), cis-β-farnesene (7.12%) in Portugal [88], chamazulene (31.48%), bisabolol and bisabolone oxide (15.71%) in Brazil [81], trans-β-pharnesene (43.5%), bisabolol oxide B (9.0%) and bisabolone oxide A (8.5%) in Serbia [84]. In the present study, camphor (16.42%), 3-carene (9.95%), β-myrcene (8.01%), α-phellandrene (4.23%) and others were the most frequent components in MCEOs derived from Taza in northern Morocco. Indeed, the variation in MCEO major chemotypes is conditioned by many environmental circumstances, such as cultivation, soil bioactive compounds percentage, storage and processing property, part (steams, leaves and flowers), time and season of collection, and genetic fluctuations [82,87,90]. Likewise, the results may also be altered by the employed techniques, in particular EO extraction and analysis methods [83].

## 4. Conclusions

Chamomile is a highly requested plant in the worldwide market, due to its high medicinal benefits and irreproachable pharmacological characteristics. In addition, there is an emerging trend of using more natural ingredients rather than chemical synthetics, since many herbal remedies are safe, easily available, healthful, and cost-effective. In this current work, we have focused on the chemical composition and identification of different pharmacological properties of *M. chamomilla* EOs, honey, and their mixture. Regarding the analysis of the phytochemicals of MCEO, among the 49 molecules identified, camphor was the major compound of our EO, followed by 3-carene, β-myrcene, and chamazulene (6.54%), as evidenced by the GC-MS method. In these results, we have also concentrated on exploring the pharmacological properties of the extracts obtained from this plant, especially their antimicrobial, antidiabetic, antioxidant, and anti-inflammatory effects. As demonstrated by the MIC and MBc methods, the EOs of the study species showed an excellent antimicrobial (*Staphylococcus aureus* ATCC 29213), antifungal (*Aspergillus niger*), and anti-yeast (*Candida albicans*) effect against all the tested strains. In terms of antidiabetic effect, the EOs exhibited the most effective levels against α-glucosidase and α-amylase. Furthermore, *M. chamomilla* EOs, honey, and its mixture exhibited antioxidant activity against the DPPH assay. The mixture was found to exhibit the best radical scavenging activity. Moreover, EOs, honey, and their mixture at a dose of 100 mg/kg exerted a high anti-inflammatory effect. We can suggest that this biological diversity of *M. chamomilla* could be ascribed to its chemical composition, containing several types of biochemical compounds. In addition, from the obtained outcomes, it is evident that the most prominent area of application of *M. chamomilla* is in the medicinal field, as demonstrated in the in vivo and in vitro models. Accordingly, the EO and extracts of the studied plant materials could be a suitable source of drug materials for the preparation of new antimicrobial, antidiabetic, antioxidant, and anti-inflammatory agents. However, the major bioactive molecules identified in the EOs of chamomile need to be investigated further, in terms of their toxicity to verify their safety and also to determine the underlying mechanisms of action. Nevertheless, despite this type of validation, we could not use these EOs because they are volatile compounds. Moreover, there is another very important perspective that requires the encapsulation of these EOs in order to provide a product in powder form that could be applied in the field of cosmetics, pharmaceutical, or food industries.

## Figures and Tables

**Table 1:** Physicochemical characteristics of *M. chamomilla* honey sample.

Sample	Color	Moisture (%)	pH	Free Acidity (meq/kg)	HMF (mg/kg)	Lactone Acidity (meq/kg)	Electrical Conductivity (mS/cm)	Density (g/mL)	Ashes (%)
*M. chamomilla*	Extra light amber	11.04 ± 0.02	4.02 ± 0.01	22.78 ± 0.01	11.04 ± 0.02	3.01 ± 0.05	0.48 ± 0.01	1.39 ± 0.01	0.36 ± 0.02

HMF: Hydroxymethylfurfural.

**Table 2:** Chemical composition of MCEO.

Compounds	RT	%
*γ*-Terpinene	2.104	2.33
Camphene	2.273	0.88
3-Carene	2.848	9.95
*β*-Myrcene	3.332	8.01
*α*–Phellandrene	3.445	4.23
(+)-4-Carene	3.614	0.61
2,4,5-tetramethyl-1,3-cyclopentadiene	3.873	5.93
Limonene	3.930	2.21
*γ*-Terpinene	4.549	0.98
*α*-Terpinolen	5.180	0.51
Linalool oxmono	6.037	0.38
Camphor	6.623	16.42
3-Methylbut-2-en-1-yl pivalate	6.893	0.53
endo-Borneol	7.152	1.76
Terpinen-4-ol	7.434	1.60
*α*-Thujenal	7.727	0.20
Terpineol	7.783	0.30
Myrtenol	7.930	0.12
Sabinol	8.031	0.41
trans-*β*-Ocimene	8.629	0.21
Thymol	10.905	1.56
*γ*-Elemene sesqui	11.457	0.15
Copaene	12.663	0.11
*β*-Cedrene	13.384	0.20
Caryophyllene	13.756	2.69
*β*-copaene	13.970	0.22
*α*-Calacorene	14.173	0.35
trans-*α*-Bergamotene	14.308	0.24
*β*-Sesquiphellandrene	14.533	3.04
Aromandendrene	14.590	0.10
Aristolochene	14.770	0.38
Germacrene	15.018	2.57
*γ*-Muurolene	15.063	0.48
*β*-Guaiene	15.142	1.20
*α*-Curcumene	15.232	0.46
*α*-Muurolene	15.412	0.23
3,6-Dihydrochamazulene	15.694	5.34
*β*-Sesquiphellandrene	15.886	1.20
Caryophyllene oxide	16.472	0.75
3-6-dihydro-Chamazulene autre	16.990	0.55
*β*-Calarene	17.136	0.52
Agarospirol	17.272	0.13
*β*-Eudesmol	17.407	1.34
Chamazulene	18.353	6.54
Dehydrochamazulene	18.748	0.40
*α*-Phellandrene	19.030	0.27
cis-.alpha.-Bergamotene	20.303	0.16
*p*-Camphorene	20.720	0.31
*p*-Cymene	21.261	1.47
Total identified compounds %		88.97
Monoterpene hydrocarbons %		37.59
Oxygenated monoterpenes %		21,72
Sesquiterpenes hydrocarbons %		14.61
Oxygenated sesquiterpenes %		2.22
Other		12.83

**Table 3:** Antioxidant effects of *M. chamomilla* honey, MCEO, and their mixture.

IC_50_ (µg/mL)	DPPH (µg/mL)	BCB (%)	FIC(µg/mL)
MCEO	533.89 ± 15.05	31.01 ± 0.09	943.61 ± 0.06
*M. chamomilla* honey	1945.38 ± 12.71	745.54 ± 08.03	1773.78 ± 02.11
Mixture	812.43 ± 05.11	326.19 ± 11.34	713.69 ± 03.02
BHT	14.24 ± 1.32	60.24 ± 0.02	42.12 ± 0.07

MCEO: *Matricaria chamomilla* essential oil; BHT: butylated hydroxytoluene; DPPH: 2,2-diphenyl-1-picrylhydrazyl; BCB: β-carotene bleaching assay; FIC: ferrous ion chelating ability.

**Table 4:** Antidiabetic effects of MCEO, *M. chamomilla* honey, and their mixture.

IC_50_ (μg/mL)	α-Amylase	α-Glucosidase
MCEO	121.44 ± 0.05	265.57 ± 0.03
*M. chamomilla honey*	981.44 ± 0.05	1351.02 ± 0.01
*Mixture*	757.23 ± 0.02	845.31 ± 0.02
Acarbose	396.42 ± 5.16	199.53 ± 1.12

**Table 5:** In vivo anti-inflammatory activity. Inhibition percentage of the left hind paw volume in rats treated with MCEO, *M. chamomilla* honey, and their mixture.

Drugs	Dose (mg/kg)	Carrageenan-Induced Hind Paw Edema Volume (mL; Mean S.E.M.) and % of Inhibition						
		**T0**	**1 h**	**% inh**	**3 h**	**%inh**	**6 h**	**% inh**
Control	-	0.87	1.45		1.67		1.87	
*M. chamomilla* honey	50	0.81	1.26	22.41%	1.22	48.75%	1.17	60.44%
	100	0.75	1.17	27.59%	1.06	61.25%	0.99	76%
MCEO	50	0.79	1.25	20.6%	1.21	47.5%	1.19	60%
	100	0.8	1.24	24.14%	1.21	48.75%	1.16	64%
MCEO and *M. chamomilla* honey mixture (1/1)	50	0.78	1.18	31.03%	1.16	52.5%	1.12	66%
	100	0.85	1.20	39.65%	1.14	63.75%	1.08	77%
Indomethacin	10	0.82	1.12	48.27%	1.17	56.25%	1.13	69%

**Table 6:** In vitro anti-inflammatory and dermatoprotective activity of MCEO, *M. chamomilla honey and mixture.*.

Assays	(IC_50_ μg/mL)			Control
	MCEO	*M. chamomilla* Honey	Mixture	Quercetin
5-Lipoxygenase	1.58 ± 0.02	5.71 ± 0.01	3.42 ± 0.02	1.02 ± 0.01
Tyrosinase	41.11 ± 0.03	81.53 ± 0.01	57.32 ± 0.03	39.28 ± 0.02

**Table 7:** The inhibitory diameters (mm) of MCEO, *M. chamomilla* honey, and their mixture.

Microorganisms	MCEO	Honey	Mixture	Chloramphenicol	Nystatin
*E. coli ATCC 25922*	16.87 ± 0.16	11.97 ± 0.11	15.37 ± 0.11	22.00 ± 0.13	NT
*P. mirabilis ATCC 25933*	19.33 ± 0.16	12.33 ± 0.11	15.83 ± 0.11	22.93 ± 0.11	NT
*S. enterica* Typhimurium *ATCC700408*	16.23 ± 0.09	11.03 ± 0.16	13.27 ± 0.16	14.57 ± 0.09	NT
*P. aeruginosa 27853*	11.20 ± 0.07	8.10 ± 0.01	10.60 ± 0.07	6.00 ± 0.01	NT
*S. aureus ATCC 29213*	22.97 ± 0.16	13.17 ± 0.09	17.67 ± 0.16	26.03 ± 0.16	NT
*L. monocytogenes ATCC 13932*	22.10 ± 0.13	13.90 ± 0.13	17.30 ± 0.13	28.73 ± 0.11	NT
*Candida albicans*	21.07 ± 0.24	12.10 ± 0.01	15.55 ± 0.25	NT	28.60 ± 0.10
*Trichophyton rubrum*	15.87 ± 0.16	8.17 ± 0.11	12.60 ± 0.13	NT	26.13 ± 0.11
*Aspergillus niger*	18.13 ± 0.18	8.13 ± 0.04	13.23 ± 0.11	NT	26.23 ± 0.11

Diameters in mm, NT: not tested.

**Table 8:** MIC and MBC of MCEO, *M. chamomilla* honey, and their mixture in percentages (*v*/*v*).

Microorganisms	Samples % (*v*/*v*)						Controls (µg/mL)	
	EO		Honey		Mixture		Chloramphenicol	Nystatin
	MIC	MBC	MIC	MBC	MIC	MBC	MIC/MBC	MIC
*E. coli* ATCC 25922	0.5	1	4	>8	2	2	4	NT
*P. mirabilis* ATCC 25933	0.5	1	4	>8	2	2	4	NT
*S*. *enterica* Typhimurium ATCC 700408	1	1	4	>8	2	2	64	NT
*Pseudomonas aeruginosa* 27853	4	>8	>8	>8	4	>8	>64	NT
*S. aureus* ATCC 29213	0.25	0.25	2	4	1	1	4	NT
*L. monocytogenes* ATCC 13932	0.25	0.25	2	4	1	1	2	NT
*Candida albicans*	0.5	NT	2	NT	1	NT	NT	4
*Trichophyton rubrum*	2	NT	8	NT	4	NT	NT	16
*Aspergillus niger*	1	NT	4	NT	2	NT	NT	16

NT: not tested.

## Data Availability

Not applicable.
